# Reduced Graphene Oxide/Polyelectrolyte Multilayers for Fast Resistive Humidity Sensing

**DOI:** 10.3390/s23041977

**Published:** 2023-02-10

**Authors:** Woojin Noh, Yuchan Go, Hyosung An

**Affiliations:** Department of Petrochemical Materials, Chonnam National University, Yeosu-si 59631, Republic of Korea

**Keywords:** humidity sensors, reduced graphene oxides, layer-by-layer assembly

## Abstract

Fast humidity sensors are of interest due to their potential application in new sensing technologies such as wearable personal healthcare and environment sensing devices. However, the realization of rapid response/recovery humidity sensors remains challenging primarily due to the sluggish adsorption/desorption of water molecules, which particularly impacts the response/recovery times. Moreover, another key factor for fast humidity sensing, namely the attainment of equal response and recovery times, has often been neglected. Herein, the layer-by-layer (LbL) assembly of a reduced graphene oxide (rGO)/polyelectrolyte is demonstrated for application in fast humidity sensors. The resulting sensors exhibit fast response and recovery times of 0.75 and 0.85 s (corresponding to times per RH range of 0.24 and 0.27 s RH^−1^, respectively), providing a difference of only 0.1 s (corresponding to 0.03 s RH^−1^). This performance exceeds that of the majority of previously reported graphene oxide (GO)- or rGO-based humidity sensors. In addition, the polyelectrolyte deposition time is shown to be key to controlling the humidity sensing kinetics. The as-developed rapid sensing system is expected to provide useful guidance for the tailorable design of fast humidity sensors.

## 1. Introduction

Humidity sensing is crucial in a wide range of fields, such as biological science [1], foods [2], electronics [3], metals [4], pharmaceuticals [5], cosmetics [6], and health monitoring systems [7]. In typical humidity sensing systems, the relative humidity (RH) is determined by converting the intensity of interaction between water molecules and sensing materials in a given environment into a measurable signal such as capacity [8,9], resistance [7,10], impedance [11,12], surface acoustic waves [13,14], light [15,16], current [17], and voltage [18,19]. Many and various materials with water-interactive properties have been studied for application in humidity sensors, including carbon-based materials [20,21,22,23], semiconductors [24,25,26,27,28,29,30,31,32,33,34], clays [35,36], and polymers [37,38,39,40,41,42]. In particular, materials based on graphene, an atomically thin, highly electrically conductive material with ultrahigh specific surface area [43,44], have shown great potential for humidity sensing [22,23,45]. However, pristine graphene exhibits poor sensitivity towards humidity due to its lack of reactive sites for interacting with polar water molecules [46]. To address this issue, graphene oxide (GO) has been investigated for its surficial oxygen-containing functional groups, such as hydroxyl, carboxyl, and epoxy groups, which enable it to interact with water molecules [9,47,48]. Furthermore, although GO is electrically insulating, it exhibits proton conductivity upon adsorption/desorption of water molecules, thus facilitating its application in capacitive or impedance-based humidity sensors [49]. The pristine GO can be converted to reduced GO (rGO) by chemical/electrochemical, thermal, microwave, or laser-induced reduction processes in order to enhance its electrical conductivity [50,51,52,53,54,55]. However, trade-offs between electrical conductivity and sensitivity are observed in rGO-based sensors due to a decrease in the number of surface functional groups during reduction [56]. To solve this problem, various research groups have explored the incorporation of graphene-based materials with other water-interactive materials such as polymers and ceramics [57,58,59,60,61,62]. However, the realization of a rapid-response/recovery humidity sensor remains challenging, primarily due to the slow adsorption/desorption of water molecules and the discrepancy between the adsorption and desorption times.

Herein, rGO/polyelectrolyte multilayer films are prepared via layer-by-layer (LbL) assembly for application in a fast humidity sensor. The sensor exhibits fast and almost identical response and recovery times, thus outperforming the majority of previously reported GO- or rGO-based humidity sensors. In addition, the humidity-sensing kinetics are shown to be controlled by the deposition time of the polyelectrolyte. Thus, the present study opens new possibilities for employing graphene-based materials in fast-response humidity sensors.

## 2. Materials and Methods

**Materials.** Poly(diallyldimethylammonium chloride) (PDAC, M_W_ = 200,000–350,000 g mol^−1^, 20 wt.% in water) was purchased from Sigma-Aldrich (Seoul, Republic of Korea). Graphite (natural, 325 mesh, 99.8%) was purchased from Alfa Aesar (Seoul, Republic of Korea). Slide glasses (SciLab, Seoul, Republic of Korea) were used as the substrates for LbL deposition. Potassium permanganate (KMnO_4_) and hydrogen peroxide (H_2_O_2_, 34.5%) were purchased from Samchun, Korea. Hydrochloric acid (HCl) was obtained from Duksan, Korea. Deionized (DI) water (resistivity ≥ 18.2 MΩ/cm, New P.Nix Power, Human Corp., Seoul, Republic of Korea) was used for all experiments.

**Synthesis of graphene oxide.** Graphene oxide was synthesized using the modified Hummers method, as described in previous work. In detail, graphite powder (3 g), concentrated H_2_SO_4_ (120 mL), and NaNO_3_ (2.5 g) were added to a round-bottomed flask in an ice bath. The mixture was stirred with a Teflon-coated magnetic stir bar at 350 rpm for 1 h, and then KMnO_4_ (15 g) was gradually added over a period of 2 h. The temperature of the mixture was kept below 20 °C by cooling it in an ice bath. The mixture was stirred at 500 rpm and 35 °C for a further 3 h. After mixing, 200 mL of DI water were added to the flask over a period of 5 h, followed by an additional 750 mL of DI water over a period of 0.5 h. Next, H_2_O_2_ (20 mL, 30 wt.%) was added dropwise, and the color of the mixture was seen to turn brown. The resulting GO dispersion was left to stand overnight. Then, the supernatant was decanted to remove the small GO particles. The sediment was then re-dispersed with dilute HCl (1 L, 3.7%) and washed three times with DI water using a centrifuge at 6000 rpm for 5 min. The resulting GO was then re-dispersed in DI water and dialyzed against DI water for 7 days to remove any residual metal ions and acids. The DI water for the dialysis was replaced with clean DI water every 24 h. The dialyzed GO dispersion (roughly 600 mL) was then mixed with DI water (2400 mL) and tip-sonicated (STH-750S, Sonictopia, Cheongju, Republic of Korea) at 375 W for 10 min to obtain exfoliated GO sheets in water.

**Preparation of the reduced graphene oxide/polyelectrolyte multilayers.** The PDAC and GO sheets were diluted to a concentration of 0.5 mg mL^−1^ in DI water to obtain a pH of 5. The glass slides were cut into 12.5 mm × 50 mm pieces, cleaned sequentially for 10 min each with water, isopropyl alcohol, and acetone via bath sonication, and then dried with air. The substrates were then subjected to plasma treatment (Harrick PDC-32G, Ithaca, NY, USA) for 3 min under vacuum, then immediately immersed in PDAC solution for the desired deposition time (2, 5, and 10 min). After rinsing three times (2, 1, and 1 min) with DI water, the substrates were immersed in GO dispersion for 10 min (unless otherwise stated), then rinsed again three times (2, 1, and 1 min) with DI water. Each cycle (immerse in PDAC/rinse/immerse in GO/rinse) is termed as a layer pair. The same procedure was repeated as necessary to obtain the desired number (0, 2, 3, 5, and 10) of layer pairs (LPs). At this stage, the electrical conductivities of the assembled films were below the detection limit of the tester because both the GO and the polyelectrolyte were electrically insulating. The GO/polyelectrolyte multilayer samples were then reduced to 220 °C under vacuum for 30 min to obtain rGO/polyelectrolyte samples with increased electrical conductivities (resistance = 10–20 MΩ/cm).

**Preparation of the humidity test cells.** The rGO/polyelectrolyte sample was cut into 5 mm × 20 mm pieces. After placing 5 mm wide tape across the rGO/polyelectrolyte film, copper wires were connected to the end of the film with silver paste (Elcoat P-100, CANS, Tokyo, Japan), followed by vacuum drying overnight. The tape was then gently removed, and the copper wires were connected to a multimeter (XDM1041 digital multimeter, Owon, Zhangzhou, China) to measure the resistance change according to the humidity. Unless stated otherwise, the 10-LP rGO/polyelectrolyte multilayer that was prepared using 2 min PDAC deposition time was used in the experimental tests.

**Characterization.** Scanning electron microscopy (SEM, SU8200, Hitachi, Japan) was used to investigate the morphologies of the multilayer films. Ultraviolet-visible (UV–vis) spectroscopy (C-7000V, Peak Instruments, Shanghai, China) was conducted over a wavelength range of 350–900 nm. The humidity response of the rGO/polyelectrolyte multilayer device was tested in a lab-built chamber at 22–23 °C. The humidity in the chamber was controlled in the test range of 20–70% RH by either bubbling air in DI water or filtering air through dried silica gel. The relative humidity in the chamber was recorded using an Arduino Uno R3 control board (Elegoo, Shenzhen, China) with a commercial humidity sensor module (AM2302 DHT22, Aosong, Guangzhou, China) having an accuracy of ±2%, while the resistance change was measured simultaneously using a multimeter (XDM1041 digital multimeter, Owon, Zhangzhou, China). The humidity environment was stabilized by waiting for 30 s after each adjustment. The humidity response and recovery times were evaluated by measuring the time required to reach a normalized response (*R*_N_) of 0.9 from an *R*_N_ of 0.1 and vice versa, with humid airflows of 0.2, 0.5, 1.0, and 2.0 Hz using an electric fan. A plot of ln[(*R*_1_ − *R*_0_)/(*R*_1_ − *R*_t_)] vs. time was then used to determine the adsorption and desorption rate constants (*k*_sorp_ and *k*_desorp_) according to the resistance change due to water adsorption or desorption from the relationship:

ln[(*R*_1_ − *R*_0_)/(*R*_1_ − *R*_t_)] = *k*_sorp_ *t* or *k*_desorp_ *t*,

where *R*_1_ and *R*_0_ are the respective final and initial resistances of the multilayer cell, and *R*_t_ is the resistance at time *t*. The sensitivity is defined by (*R*_1_ − *R*_0_)/(*RH*_0_ − *RH*_1_), where *RH*_0_ and *RH*_1_ are the respective initial and final RHs (%) of the multilayer cell. The limit of detection (LOD) is defined as the lowest detectable humidity and is calculated from the relation *LOD* = 3*SD*/*m* [63], where *SD* is the standard deviation of the response, and *m* is the slope of the calibration curve.

## 3. Results

**Figure 1:**
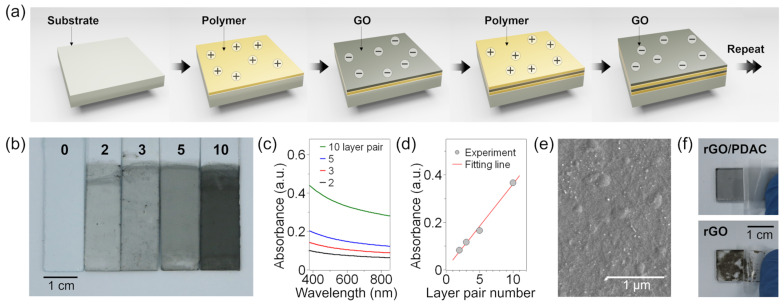
Characterization of the as-assembled reduced graphene oxide (rGO)/polyelectrolyte multilayers: (**a**) a schematic diagram of the layer-by-layer (LbL) assembly technique; (**b**) a digital image of the rGO/polyelectrolyte multilayer films with 0, 2, 3, 5, and 10 layer pairs (LPs) on glass substrates, prepared by the sequential adsorption of PDAC (10 min) and GO (2 min), followed by thermal reduction; (**c**) the corresponding UV–vis absorption spectra; (**d**) the UV-vis absorbance values at 550 nm vs. the number of layer pairs (*R*^2^ = 0.992); (**e**) an SEM image of the 10-LP rGO/polyelectrolyte multilayer film; (**f**) photographic images showing the adhesion testing of the rGO/polyelectrolyte multilayer film (top) and the rGO sheet (bottom) with 3 M Scotch tape.

The graphene oxide sheets and the rGO/polyelectrolyte multilayers on glass substrates were synthesized as described in the Materials and Methods section and are shown schematically in Figure 1a. Previous studies demonstrated that an aqueous dispersion of GO is stable at a pH of 4 due to the negatively-charged particle surfaces, with zeta potentials typically ranging from −30 to −35 mV [64,65], while the PDAC (conc. = 0.5 mg/mL, pH = 5) is positively charged (+18 mV). As shown in Figure 1b, the multilayers become visibly darker as the number of layer pairs (LbL cycles) increases from 0 to 10. Moreover, broad adsorptions at 550 nm in the UV-vis spectra are seen to increase linearly with the number of layer pairs (Figure 1c,d), thus suggesting a linear increase in film thickness. Note that the broad adsorption is attributed to the rGO [66], while the PDAC barely absorbs wavelengths of over 400 nm [67]. Meanwhile, the SEM image of the rGO/polyelectrolyte multilayer film in Figure 1e reveals a dense coverage of the film on the surface. In addition, when an adhesion test was performed using 3M tape, the as-fabricated film exhibited strong mechanical integrity, while a comparable rGO sample on a glass substrate did not (Figure 1f). This comparison highlights the importance of the attractive electrostatic interactions between the GO sheets and the polyelectrolyte for the successful assembly of rGO/polyelectrolyte multilayers, as reported elsewhere [68,69].

**Figure 2:**
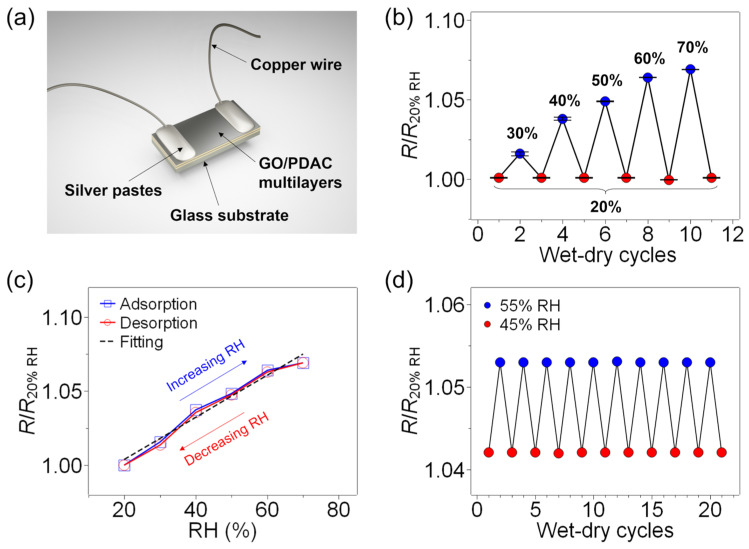
The humidity-sensing performance of the rGO/polyelectrolyte multilayers: (**a**) a schematic diagram of the rGO/polyelectrolyte multilayer device on a glass slide with copper wire connections for current collection; (**b**) the effect of increasing RH (%) upon the normalized resistance (*R*/*R*_20%_), where *RH*_20%_ indicates an RH of 20%; (**c**) the hysteresis curves, for which a linear fitting yields *y* = 0.00143*x* + 0.975, with *R*^2^ = 0.974; (**d**) the cycling performance when alternating between 40% and 50% RH at room temperature (22–23 °C).

The preparation of the humidity test cell is described in the Materials and Methods section, and the completed cell is shown schematically in Figure 2a. The effect of varying the relative humidity (RH) upon the resistance of the 10-LP rGO/polyelectrolyte multilayer with the 2 min PDAC deposition time is shown in Figure 2b. Here, each red point represents an RH of 20%, while the blue points represent increased RH values of 30–70%. Thus, the resistance is seen to increase by a total of 7% as the RH increases from 20% to 70% (providing a sensitivity of 14 kΩ/%RH) and is fully recovered upon returning the RH to 20%. The humidity sensing performance is further demonstrated in Figure 2c, with little hysteresis. From this result, the LOD is extracted as 10.3% RH. Moreover, the multilayer film shows a stable resistance response during 21 cycles (2 h total) of RH, switching from 40% to 50% (Figure 2d).

**Figure 3:**
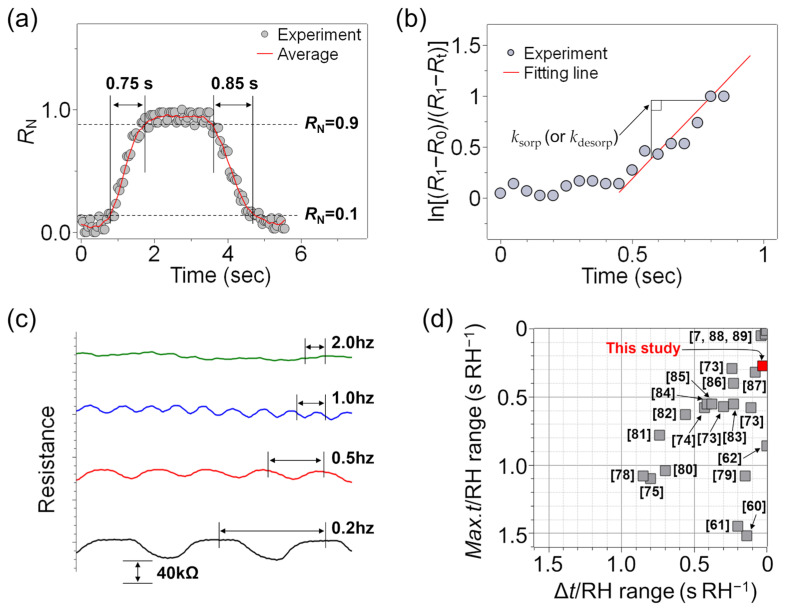
The humidity response and recovery times of the 10-LP rGO/polyelectrolyte multilayer that was prepared with a 2 min PDAC deposition time: (**a**) the normalized response (*R*_N_); (**b**) the plot of ln[(*R*_1_ − *R*_0_)/(*R*_1_ − *R*_t_)] vs. time for determining the rate constants (*k*_sorp_ or *k*_desorp_); (**c**) the response/recovery in the presence of humid airflows with various frequencies; (**d**) a plot of the maximum response/recovery time (*Max.t*) vs. the absolute difference between the recovery and response times (Δ*t*) (each normalized according to the RH interval tested) for the as-fabricated and previously reported sensors.

The response and recovery times of the multilayers were evaluated with the interruption of the humid airflow at a frequency of 0.2 Hz in the RH range of 57 to 61% using an electric fan. The results in Figure 3a indicate that the rGO/polyelectrolyte multilayer provides response and recovery times of 750 and 850 ms, respectively. For a fair comparison with the results of previously reported humidity sensors having widely different RH ranges, these values are converted to the time per RH range tested (s RH^−1^) as a figure of merit on the assumption that the humidity response is linear [7]. This provides response and recovery values of 0.24 and 0.27 s RH^−1^, respectively, for the present work. Further, the plot of ln[(*R*_1_ − *R*_0_)/(*R*_1_ − *R*_t_)] against time in Figure 3b reveals that the as-fabricated sample has adsorption and desorption rate constants of 2.7 and 2.6 s^−1^, respectively, which are comparable with those obtained in previous work on ultrafast humidity sensors [7].

The above results clearly demonstrate the reversibility of the water–molecule adsorption/desorption behavior of the multilayer film according to the increase and decrease in humidity and are consistent with the results of previous studies [7,70]. Thus, the response/recovery behavior of the humidity sensor might be explained by the previously established mechanism involving changes in the interlayer spacing [7] or by a decrease in conductivity due to the interactions between the water molecules and the surface functional groups of the rGO, which would alter the electronic structure of the rGO itself [71,72]. Hence, to explore the potential tunability of the sensing kinetics due to the ability of PDAC to absorb and desorb water, the adsorption and desorption rate constants were calculated for the PDAC deposition times of 2, 5, and 10 min, providing *k*_sorp_ values of 2.7, 2.0, and 1.7 s^−1^, respectively. This indicates that an increase in the PDAC deposition time leads to a decrease in the rate constant, thus confirming the crucial role of PDAC in humidity sensing. Based on these results, it appears that the change in interlayer spacing is the predominant response/recovery mechanism. Moreover, the as-fabricated sensors exhibit fast humidity sensing capabilities at various airflow modulation frequencies of up to 1.0 Hz (Figure 3c), thus demonstrating their potential use in applications involving dynamic changes in humidity, such as human respiration monitoring sensors.

As noted in the Introduction, achieving equal response and recovery times is another key requirement that has often been ignored, especially for a rapid response towards dynamic changes in humidity. In the present work, the rGO/polyelectrolyte multilayer exhibits an absolute difference between the recovery and response times (Δ*t*_response-recovery_) of 0.1 s (corresponding to 0.03 s RH^−1^), thus indicating that the recovery and response times are nearly identical. This result is compared with those of previous studies in Table 1 and Figure 3d. This provides an important performance indicator, with smaller values of both the maximum response/recovery time per RH range and the absolute difference between the recovery and response times per RH range being beneficial for fast-response humidity sensors. Thus, the present results indicate that the as-fabricated rGO/polyelectrolyte multilayer outperforms the majority of previously reported graphene-based humidity sensors such as GOs [73], rGO/Ag [74], GO/TiO_2_ [62], GO/MoS_2_ [59], GO/WS_2_ [61], graphene quantum dots [75], rGO/lignosulfonate [58], etc.

## 4. Conclusions

Herein, a fast humidity sensor was demonstrated using reduced graphene oxide (rGO)/polyelectrolyte multilayers prepared via layer-by-layer (LbL) assembly. The as-fabricated sensors exhibited fast and nearly identical response and recovery times, exceeding those of most previously reported GO- or rGO-based humidity sensors. In addition, the deposition time of the polyelectrolyte was shown to be a key factor in controlling the humidity sensing kinetics, with a short polymer deposition time leading to a faster response. Hence, in future work, the humidity response of the sensor might be further modulated and intensified by optimizing the LbL assembly process in order to identify the minimum amount of PDAC needed to form the multilayer structure while achieving the fastest possible response/recovery times. In addition, the underlying mechanism of the reversible resistance change must be elucidated, with two possible contributions being a change in the interlayer spacing due to the water-sensitive polymer [7,70] and a change in the electronic structure of the GO sheet due to the interactions between polar water molecules and the terminal groups [72,90]. Finally, an obvious future research direction is the development of wearable personal healthcare and environment sensing devices based on the humidity sensor materials developed herein.

## Figures and Tables

**Table 1:** The performance characteristics of various resistive humidity sensors.

Materials	Max. *t*/RH-Range (s RH^−1^)	Δ*t*/RH-Range (s RH^−1^)	*t*_recovery_/RH-Range (s RH^−1^)	*t*_response_/RH-Range (s RH^−1^)	Ref.
MoS_2_	10.00	7.00	11.00	4.00	[76]
rGO/hydrophobin	8.00	2.80	8.00	5.20	[57]
rGO/lignosulfonate	6.70	3.40	3.30	6.70	[58]
rGO/MoS_2_	5.06	4.46	5.06	0.60	[59]
rGO/lignosulfonate	4.10	3.48	0.62	4.10	[58]
N-doped TiO_2_	3.69	3.47	3.69	0.22	[77]
rGO/PDAC	1.52	0.14	1.37	1.52	[60]
GO/WS_2_	1.45	0.20	1.45	1.25	[61]
Graphene quantum dots	1.10	0.80	1.10	0.30	[75]
SiO_2_/GO-NH_2_	1.08	0.85	1.08	0.23	[78]
GO/MoS_2_	1.08	0.15	0.93	1.08	[79]
rGO/WS_2_	1.04	0.70	1.04	0.34	[80]
GO/TiO_2_	0.86	0.00	0.86	0.86	[62]
rGO/silk	0.78	0.74	0.78	0.04	[81]
ZnO	0.63	0.56	0.08	0.63	[82]
rGO/Ag	0.58	0.43	0.15	0.58	[74]
B-doped GO	0.58	0.11	0.58	0.47	[73]
GO	0.57	0.30	0.57	0.27	[73]
Graphene-carbon ink	0.55	0.41	0.14	0.55	[83]
PVDF/ZnO	0.55	0.23	0.55	0.32	[84]
Black P/graphene	0.55	0.38	0.55	0.16	[85]
Polyvinyl alcohol	0.40	0.23	0.40	0.17	[86]
SnO_x_/carbon fibers/graphene	0.32	0.08	0.24	0.32	[87]
Li-doped GO	0.29	0.24	0.29	0.05	[73]
Fe-doped SnO_2_	0.05	0.04	0.05	0.01	[88]
rGO/PVP	0.04	0.01	0.04	0.03	[89]
MXene/PDAC	0.01	0.005	0.01	0.005	[7]
rGO/PDAC	0.27	0.03	0.27	0.24	This study

## Data Availability

Not applicable.
